# Chronic Traumatic Tibialis Posterior Tendon Rupture in an Adolescent With Progressive Planovalgus Deformity: A Case Report

**DOI:** 10.7759/cureus.117763

**Published:** 2026-10-11

**Authors:** Mahmoud Sbaihat, Laith Hseinat, Mohammad Raed, Qusai Alazzam, Refat AlRawashdeh, Mohammad Al-Dweeri, Shujaa Al-Jarajreh, Qusai Alqarqaz, Marwan Abo Hashim, Ahmed Almarzouq

**Affiliations:** 1 Orthopaedics and Traumatology, Jordanian Royal Medical Services, Amman, JOR; 2 Orthopaedics, Jordanian Royal Medical Services, Amman, JOR; 3 Orthopaedics, Yarmouk University, Irbid, JOR; 4 Orthopaedics and Traumatology, King Hussein Medical City, Amman, JOR; 5 Orthopaedics, Ministry of Health, Amman, JOR

**Keywords:** adolescent, adolescent planovalgus deformity, flexor hallucis longus tendon transfer, pediatric flatfoot, planovalgus deformity, posterior tibial tendon rupture, talocalcaneal coalition, tibialis posterior tendon

## Abstract

Posterior tibial tendon rupture is rare in children and adolescents and may be overlooked following an ankle injury. Delayed diagnosis can result in persistent tendon dysfunction and progressive planovalgus deformity. A 13-year-old boy developed progressive planovalgus deformity following an eversion and external-rotation injury to the foot and ankle. Initial management included shoe modification; however, the deformity and functional impairment persisted. Clinical examination demonstrated loss of the medial longitudinal arch, impaired posterior tibial tendon function, and inability to perform a single-leg heel rise. MRI showed nonvisualization of the posterior tibial tendon at the ankle with tibialis posterior muscle atrophy, supporting chronic complete tendon rupture. Due to the chronic nature of the lesion and absence of usable tendon tissue for primary repair, reconstruction was performed using flexor hallucis longus tendon transfer, triple percutaneous tendo-Achilles lengthening, medial translation calcaneal osteotomy, and lateral column lengthening. At three years and nine months of follow-up, the patient was pain-free, had near-normal walking and activity levels, satisfactory heel alignment, near-normal restoration of the medial longitudinal arch, and was able to perform a single-leg heel rise. Chronic posterior tibial tendon rupture should be considered in adolescents who develop progressive flatfoot following an ankle injury, particularly when posterior tibial tendon function is impaired. MRI can assist in identifying chronic tendon disruption when the diagnosis is uncertain. In chronic cases where primary tendon repair is not feasible, tendon transfer combined with appropriate correction of the associated planovalgus deformity may provide satisfactory long-term functional outcomes.

## Introduction

The posterior tibial tendon (PTT) plays an important role in maintaining the medial longitudinal arch and dynamically stabilizing the hindfoot during gait. It contributes to inversion and plantarflexion of the foot and provides dynamic support to the medial side of the foot during weight bearing [1]. Through its course behind the medial malleolus and insertion into the navicular and adjacent structures, the PTT acts as an important dynamic stabilizer of the medial column of the foot. Dysfunction or rupture of the PTT can therefore impair normal foot mechanics and result in progressive collapse of the medial longitudinal arch, hindfoot valgus, and forefoot abduction, ultimately producing an acquired planovalgus deformity [2].

PTT insufficiency is predominantly encountered in adults and is commonly associated with degenerative or chronic overuse-related pathology. In contrast, traumatic PTT rupture in children and adolescents is rare and may be overlooked because of its low incidence and the relatively broad differential diagnosis of pediatric flatfoot [3]. Pediatric PTT injuries may initially go unrecognized and subsequently present with acquired unilateral flatfoot or valgus deformity. Progressive deformity following an injury should therefore raise suspicion for an underlying soft-tissue injury, particularly when there is persistent weakness of tibialis posterior function or inability to perform a single-leg heel rise [4,5].

Pediatric flatfoot has a broad differential diagnosis, and assessment should consider the flexibility of the deformity, hindfoot alignment, subtalar motion, and potential underlying structural or soft-tissue abnormalities. Careful clinical assessment is therefore important when the presentation is acquired, asymmetric, or associated with a history of trauma. Distinguishing between flexible developmental flatfoot and an acquired or progressive deformity is particularly relevant because the underlying pathology may influence both the diagnostic workup and subsequent treatment [6].

We report the case of a 13-year-old boy who developed progressive planovalgus following an eversion and external-rotation injury. Despite initial conservative management, the deformity and functional impairment progressed, with persistent clinical evidence of tibialis posterior dysfunction. MRI demonstrated complete PTT disruption with tibialis posterior muscle atrophy, and intraoperative exploration confirmed chronic absence of a reconstructible tendon. Reconstruction was performed using flexor hallucis longus (FHL) transfer, tendo-Achilles lengthening, and calcaneal osteotomies. The patient demonstrated a favorable clinical and functional outcome at At three years and nine months of follow-up, with restoration of medial arch alignment, improved heel-rise function, and no clinically significant functional limitation.

## Case presentation

In early 2022, a 13-year-old boy with no significant medical or surgical history sustained a fall involving an eversion and external-rotation mechanism of the foot and ankle. Following the injury, the patient progressively developed a visible flatfoot deformity, clinically characterized as pes planovalgus. Following the injury, the patient was evaluated at several healthcare facilities because of the progressive and persistent foot deformity and associated functional impairment.

Clinical examination demonstrated loss of the medial longitudinal arch and impaired PTT function. The patient was unable to perform a single-leg heel rise on the affected side. On weight-bearing examination, forefoot abduction and hindfoot valgus were evident, with a positive too-many-toes sign. The planovalgus deformity was passively correctable in all components, indicating a flexible deformity, and the patient did not report pain associated with the deformity. Passive ankle dorsiflexion reached the neutral position, with no fixed equinus contracture, and the Silfverskiöld test [7] was negative. Passive subtalar motion was full and painless. Active eversion was preserved, whereas active inversion was absent. FHL and peroneal tendon function were intact, and the neurovascular examination was normal. These findings supported significant PTT dysfunction associated with a flexible planovalgus deformity (Figure 1).

**Figure 1:**
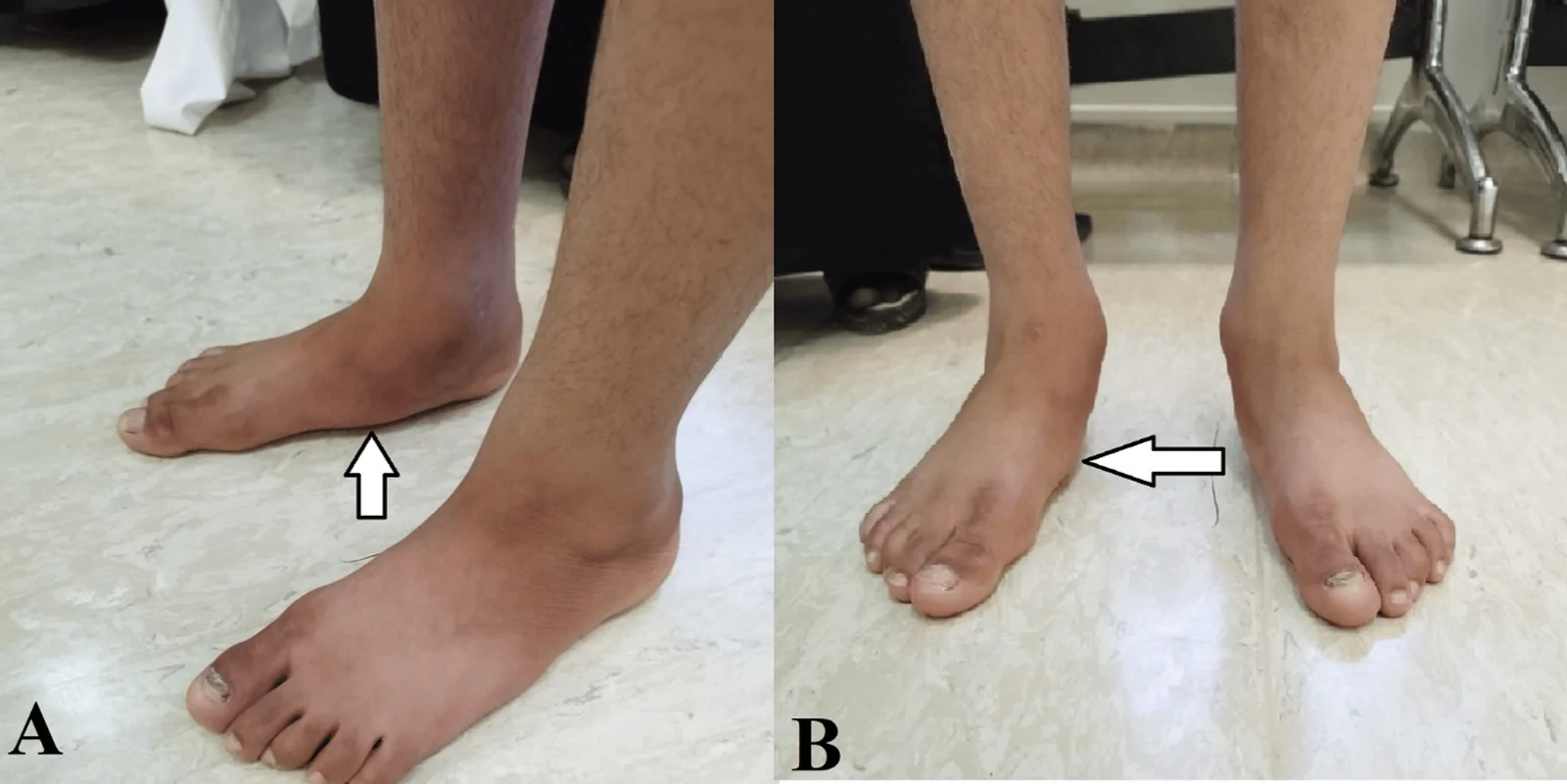
Initial clinical presentation of right pes planovalgus deformity Initial gross images of the right foot showing complete loss of the medial longitudinal arch (arrow), with a flatfoot deformity, hindfoot valgus, and forefoot abduction (pes planovalgus deformity).

Despite conservative management, the clinical deformity and functional deficit persisted. Several months later, MRI of the affected leg, foot, and ankle was obtained for further evaluation. Computed tomography of the foot and ankle was also reviewed to assess the bony architecture and potential structural causes of the planovalgus deformity. On subsequent review by a musculoskeletal radiologist, no talocalcaneal coalition was identified. MRI demonstrated that the PTT was not visualized at the level of the ankle joint. Atrophic changes were also identified in the tibialis posterior muscle, findings consistent with chronic complete disruption of the tendon (Figures 2, 3).

**Figure 2:**
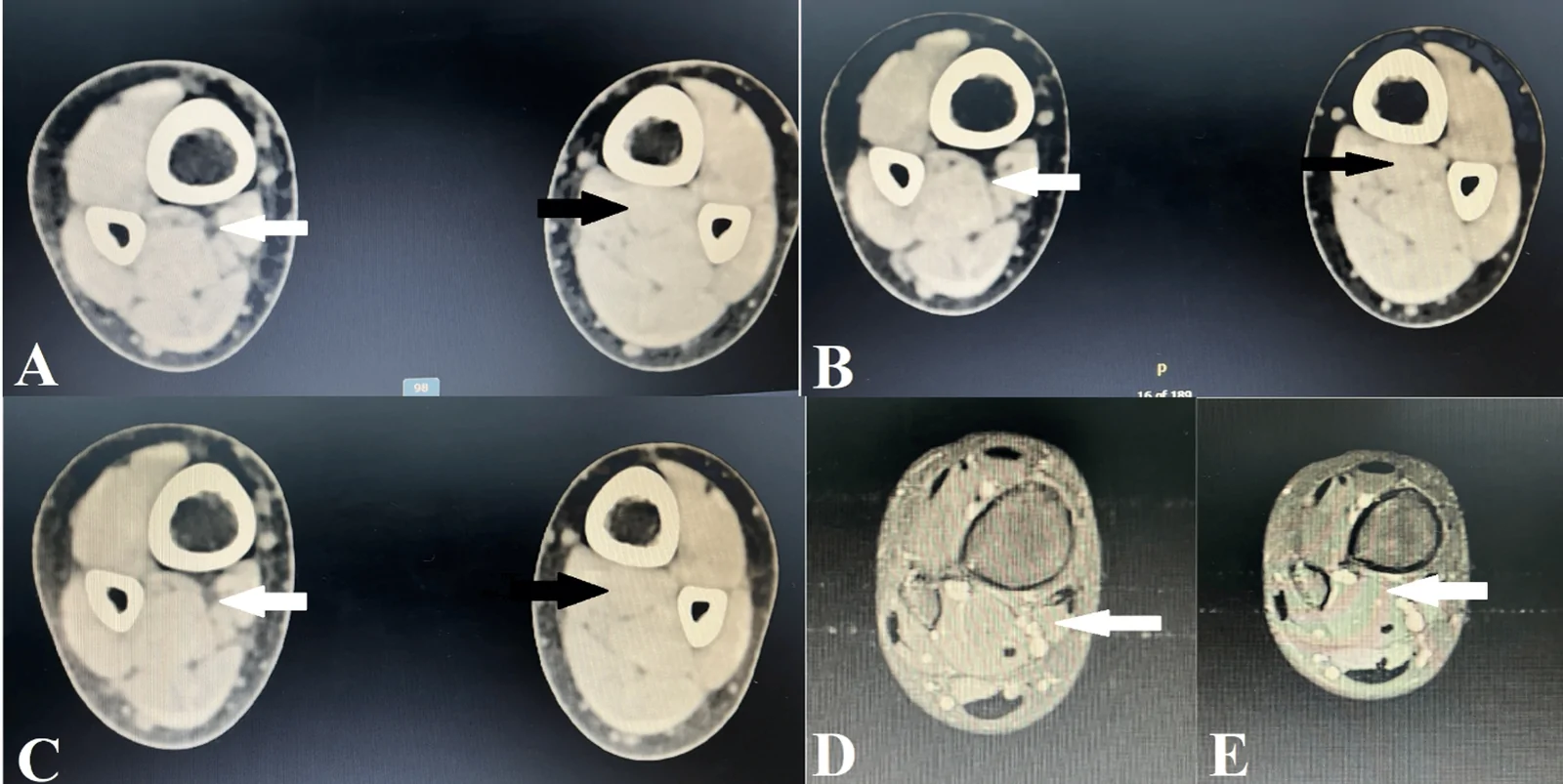
MRI findings of chronic tibialis posterior tendon rupture (A–C) T1-weighted axial MRI images of the right lower leg demonstrating marked atrophy of the tibialis posterior muscle, extending more than 8 cm proximally from the ankle joint level, compared with preserved muscle bulk on the contralateral side; (D–E) T2-weighted axial MRI images of the right ankle demonstrating absence of the tibialis posterior tendon immediately proximal to the ankle joint level, before its expected course posterior to the medial malleolus.

**Figure 3:**
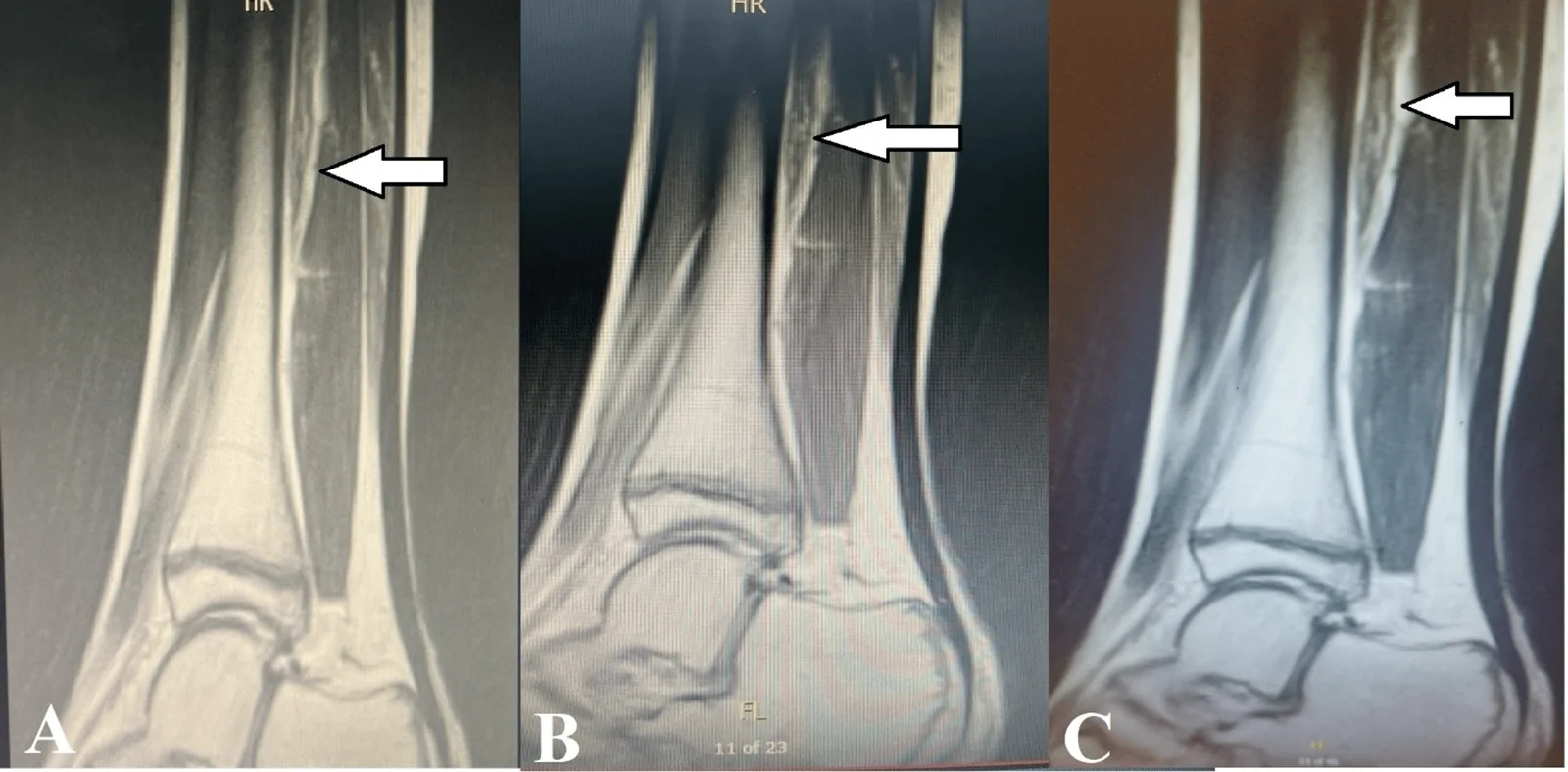
Sagittal MRI findings of tibialis posterior muscle atrophy T1-weighted sagittal MRI images showing atrophy of the tibialis posterior muscle with fatty degeneration of the muscle bulk at the level of the mid-leg (arrow).

No ankle joint effusion was identified. The combination of the clinical loss of tibialis posterior function, inability to perform a single-leg heel rise, and MRI evidence of tendon discontinuity with muscle atrophy supported the diagnosis of chronic complete PTT rupture.

Given the chronic nature of the tendon injury and the persistent functional deficit, surgical reconstruction was planned to address both the absent PTT function and the associated flexible planovalgus deformity. Preoperative examination demonstrated a flexible and painless planovalgus deformity with forefoot abduction and hindfoot valgus on weight-bearing examination, which was fully passively correctable. The patient had limited ankle dorsiflexion to the neutral position, while the Silfverskiöld test was negative. He was unable to perform a single-leg heel rise, consistent with significant PTT dysfunction. FHL and peroneal tendon function were preserved, and neurovascular examination was normal.

Because MRI demonstrated chronic absence of the PTT with associated tibialis posterior muscle atrophy, and because a reconstructible tendon stump was not expected to be available, FHL tendon transfer was selected to restore dynamic medial support. The medializing calcaneal osteotomy was performed to correct the clinically evident hindfoot valgus and restore the hindfoot mechanical alignment. Lateral column lengthening was selected to address the associated forefoot abduction and planovalgus component. A triple percutaneous tendo-Achilles lengthening was then performed using the Hoke triple-hemisection technique [8] to address the limited ankle dorsiflexion identified preoperatively.

The magnitude of the bony correction was individualized according to the clinical deformity and the intraoperative correction obtained rather than according to a predefined radiographic threshold. Approximately 10 mm of medial translation was achieved with the calcaneal osteotomy, and approximately 10 mm of lateral column lengthening was performed. Following correction, the alignment and position of the osteotomies were assessed clinically and fluoroscopically, and satisfactory correction was confirmed before fixation (Figure 4).

**Figure 4:**
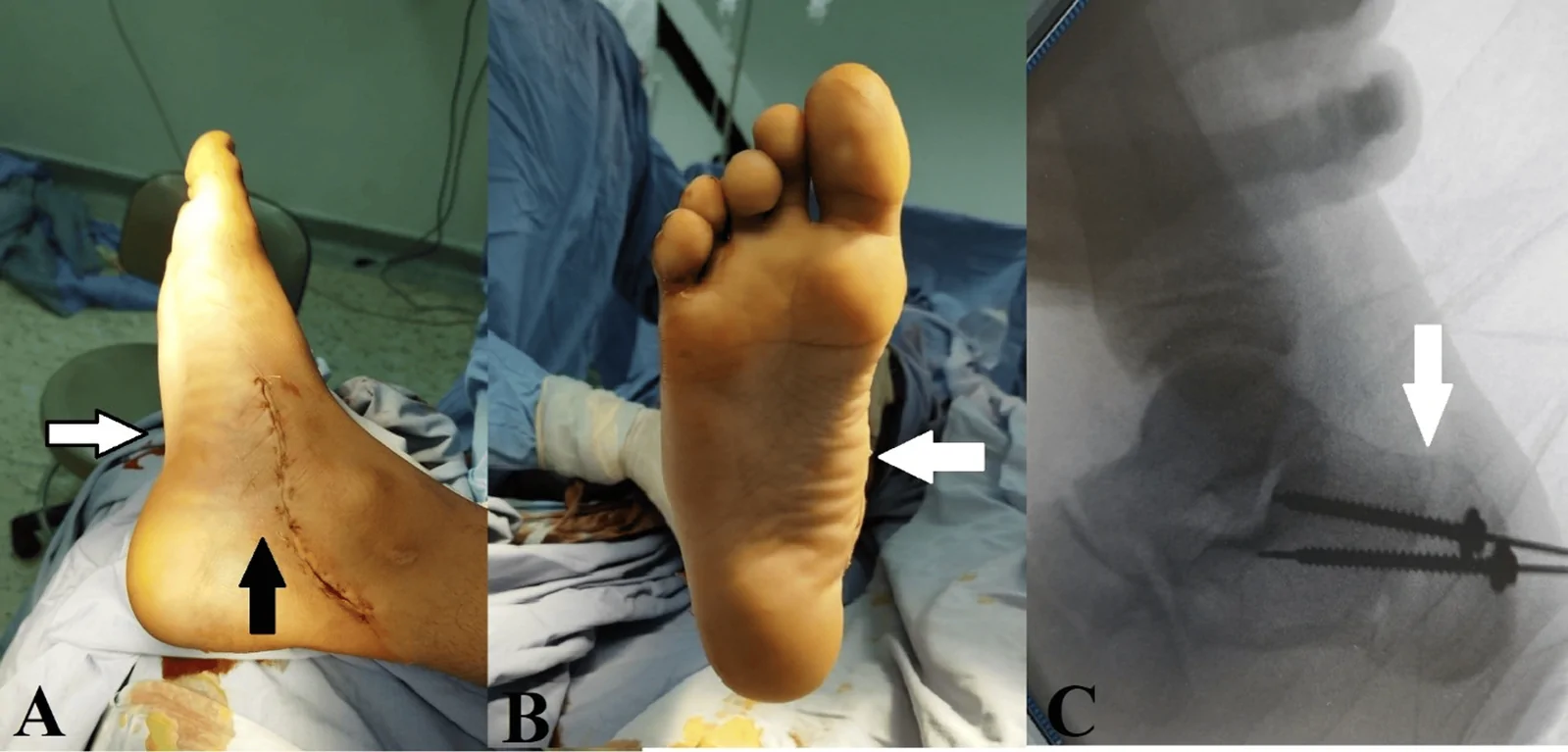
Intraoperative correction of the pes planovalgus deformity (A–B) Intraoperative gross images demonstrating the corrected medial foot contour with improved visualization of the medial longitudinal arch (white arrow) and the medial approach incision to the hindfoot (black arrow); (C) Lateral-view intraoperative fluoroscopic image demonstrating the medial calcaneal sliding osteotomy (white arrow) and fixation of the osteotomy with cannulated screws.

Through a sinus tarsi approach, the peroneal tendons were retracted, the anterior calcaneal region was exposed. A lateral column lengthening osteotomy of approximately 10 mm was performed and stabilized using a four-hole foot fixation system. The correction and implant position were assessed fluoroscopically (Figure 5).

**Figure 5:**
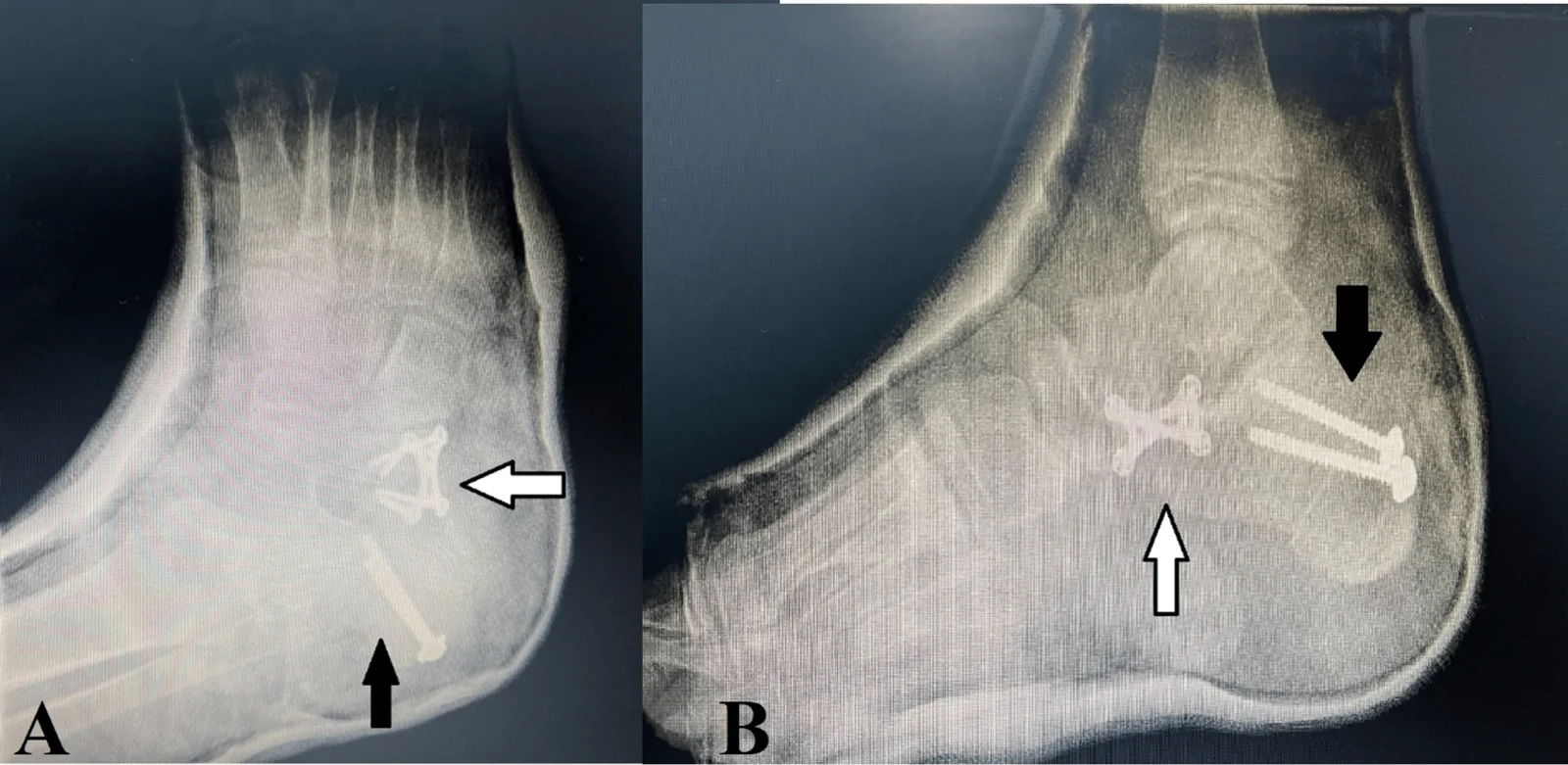
Initial postoperative radiographic assessment Postoperative weight-bearing radiographs of the foot, including oblique (A) and lateral (B) views, demonstrating correction of the planovalgus alignment following medializing calcaneal osteotomy and lateral column lengthening. The lateral view demonstrates improved calcaneal pitch following correction, with satisfactory position of the osteotomies and implants.

Attention was then directed to the posteromedial aspect of the ankle and through medial approach to hindfoot. Following layer-by-layer dissection and identification and protection of the neurovascular bundle, the expected PTT tunnel was explored. The tunnel was empty, and the proximal PTT stump could not be identified. Further distal dissection to the navicular tuberosity similarly demonstrated no identifiable PTT remnants. The distal PTT sheath collapsed. These findings confirmed a chronic tendon lesion that was not amenable to primary repair. The FHL tendon was identified through the posteromedial approach and harvested approximately 2 cm distal to the navicular level and proximal to the Knot of Henry. The tendon was prepared using a Krackow suture technique and mobilized for transfer. Two transosseous tunnels were created in the navicular using a drill bit. The prepared FHL tendon was passed into the navicular tunnels and secured to the navicular bone using No. 1 Ethibond sutures. The transfer was tensioned with the ankle maintained in a neutral dorsiflexion position. No additional tendon graft or tendon augmentation was used.

Hemostasis was secured and the wounds were closed in layers. Sterile dressings were applied, followed by a below-knee plaster-of-Paris cast. Distal perfusion was satisfactory at completion of the procedure. Following surgery, the limb was immobilized in a below-knee cast (Figure 5).

Postoperatively, the patient was immobilized in a below-knee cast for six weeks. Following cast removal, range-of-motion and proprioception exercises were initiated, together with partial weight-bearing for two weeks. During the subsequent four weeks, progressive stretching exercises targeting the FHL and tendo-Achilles were introduced, with progression to weight-bearing as tolerated. After radiographic evidence of healing of the calcaneal osteotomy sites was confirmed, the patient progressed to full weight-bearing and strengthening exercises, together with continued stretching and proprioceptive training.

The patient returned to sports approximately six months after surgery. Return to football was permitted after satisfactory functional recovery, including minimal or no pain during daily activities, tolerance of full daily activities and activities of daily living, adequate tolerance of impact and plyometric activities, and restoration of full knee and ankle range of motion and mobility.

Following completion of the reconstruction, the foot demonstrated satisfactory correction of the previously observed planovalgus alignment, with restoration of the medial longitudinal arch and correction of the hindfoot valgus and forefoot abduction. The correction and implant positions were assessed clinically and with intraoperative fluoroscopy before completion of fixation.

At three years and nine months after reconstruction, when the patient was 16 years and seven months old, he reported no pain and had returned to his previous level of walking, daily activities, school activities, and recreational and football participation. Clinical assessment demonstrated satisfactory heel alignment and near-normal restoration of the medial longitudinal arch. Importantly, the patient was able to perform a single-leg heel rise, with normal inversion of the heel during the maneuver, which had been impossible before surgery (Figure 6).

**Figure 6:**
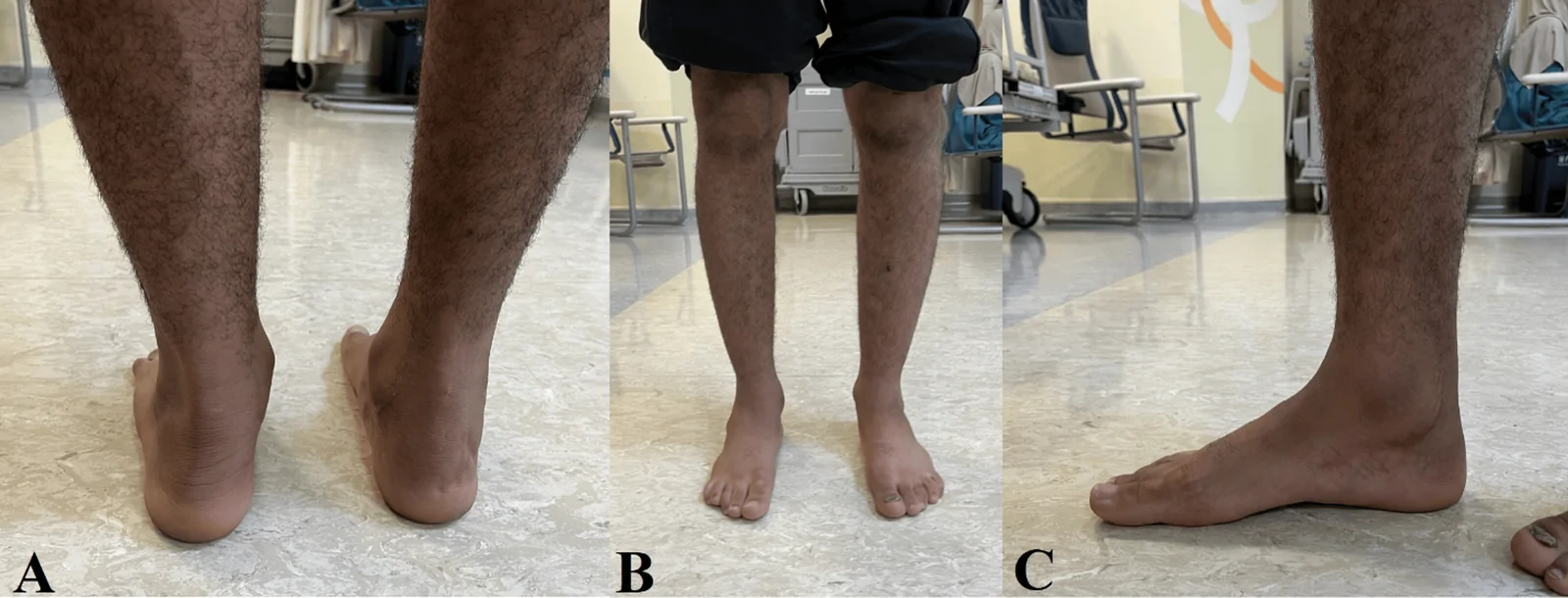
Clinical outcome at three years and nine months of follow-up Gross images at 3–4-year follow-up demonstrating healed surgical incisions, maintenance of the restored medial longitudinal arch, neutral hindfoot alignment, and restoration of the neutral foot axis.

He reported no clinically significant functional limitation or meaningful reduction in his usual activity level at the latest follow-up. Radiographs demonstrated healed calcaneal osteotomies with maintained correction, no radiographic evidence of subtalar or calcaneocuboid joint arthritis, and retained implants (Figure 7).

**Figure 7:**
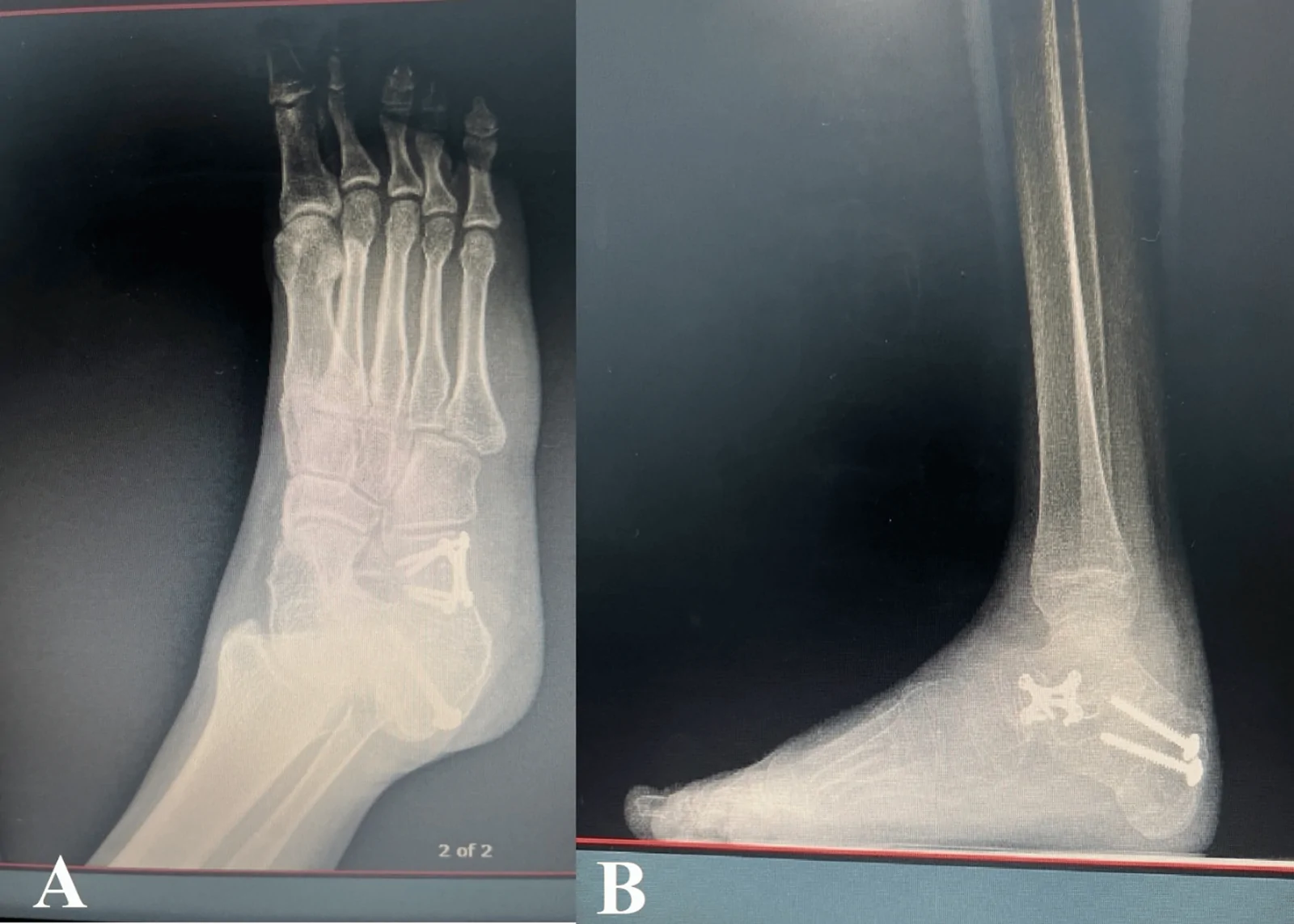
Radiographic outcome at the three-year nine-month follow-up Weight-bearing foot radiographs at 3 years and 9 months after reconstruction: oblique (A) and lateral (B) views, demonstrating maintained correction and retained metal implants. No radiographic evidence of subtalar or calcaneocuboid degenerative changes was identified.

No postoperative complications were identified during the three-year and nine-month follow-up. Specifically, there were no infections, wound complications, nerve or vascular injuries, delayed union or nonunion, hardware irritation, recurrent deformity, tendon transfer failure, limitation of FHL function, subtalar stiffness, or calcaneocuboid joint symptoms, and no revision surgery or other reoperation was required. The clinical timeline is given in Table 1.

**Table 1:** Clinical timeline of the patient's presentation, diagnosis, treatment, rehabilitation, and follow-up PTT: Posterior tibial tendon.

Time point	Clinical course / Findings	Investigation / Management
Early 2022 – Initial injury	13-year-old boy sustained a fall involving an eversion and external-rotation mechanism of the foot and ankle. No pre-injury foot deformity or related complaints were reported.	Initial medical evaluation; conservative management was instituted.
Following months	Progressive development of a visible unilateral planovalgus/flatfoot deformity with persistent functional deficit.	Multiple clinical evaluations; shoe modification was attempted.
Subsequent evaluation	Persistent loss of the medial longitudinal arch and impaired posterior tibial tendon function. The patient was unable to perform a single-leg heel rise. Weight-bearing examination demonstrated forefoot abduction and hindfoot valgus.	Further clinical assessment and advanced imaging were planned.
MRI evaluation	PTT was not visualized at the ankle, with associated tibialis posterior muscle atrophy, consistent with chronic complete tendon disruption.	MRI reviewed and confirmed by a musculoskeletal radiologist.
Preoperative assessment	Flexible and painless planovalgus deformity with hindfoot valgus and forefoot abduction. Passive subtalar motion was full and painless; active inversion was absent and eversion was preserved. Ankle dorsiflexion reached neutral, with a negative Silfverskiöld test.	Diagnosis of chronic complete PTT rupture with associated flexible planovalgus deformity.
Surgical treatment	Chronic PTT was not amenable to primary repair because no identifiable proximal or distal tendon remnant was found.	FHL tendon transfer, triple percutaneous tendo-Achilles lengthening, 10-mm medializing calcaneal osteotomy, and approximately 10-mm lateral column lengthening were performed.
First 6 weeks postoperatively	Foot maintained in postoperative immobilization.	Below-knee cast for 6 weeks.
6–8 weeks postoperatively	Initiation of functional rehabilitation following cast removal.	Range-of-motion and proprioception exercises; partial weight-bearing for 2 weeks.
8–12 weeks postoperatively	Progressive restoration of mobility and weight-bearing.	FHL and tendo-Achilles stretching with progression to weight-bearing as tolerated.
After radiographic osteotomy healing	Progressive strengthening and full weight-bearing.	Full weight-bearing, stretching, strengthening, and proprioceptive exercises.
Approximately 6 months	Functional recovery sufficient for return to sports.	Return to football after satisfactory pain control, daily activity, impact/linear plyometric tolerance, and functional mobility.
3 years and 9 months after reconstruction	At age 16 years and 7 months, the patient was pain-free and had returned to his previous level of walking, daily activities, school activities, recreational activities, and football participation. Single-leg heel rise was restored with normal heel inversion.	Clinical examination demonstrated satisfactory heel alignment and near-normal restoration of the medial longitudinal arch.
Final radiographic follow-up – 3 years and 9 months	Maintained correction without clinically significant functional limitation.	Radiographs demonstrated healed calcaneal osteotomies, maintained correction, retained implants, and no radiographic evidence of subtalar or calcaneocuboid arthritis.

Informed consent was obtained from the patient's parents for publication of the case details, including radiological images, intraoperative photographs, follow-up photographs, and intraoperative fluoroscopic images.

## Discussion

Traumatic rupture of the PTT is an uncommon cause of acquired flatfoot in children and adolescents, and diagnosis may be delayed because pediatric flatfoot has several structural and developmental causes. Pediatric PTT injury may remain unrecognized for a prolonged period and later present with progressive unilateral flatfoot or planovalgus deformity. Chronic tendon deficiency can therefore become apparent only after the development of a visible deformity and functional impairment [9,10]. In the present case, the patient had no reported foot deformity, posterior tibial tendon dysfunction, or related complaints before the injury. Following the reported closed twisting injury involving eversion and external rotation, he subsequently developed progressive planovalgus deformity and loss of posterior tibial tendon function. The contralateral foot remained clinically normal without comparable deformity or functional impairment. MRI and CT were reviewed with a musculoskeletal radiologist; no talocalcaneal coalition was identified, and MRI confirmed chronic PTT disruption with tibialis posterior muscle atrophy. However, because early post-injury imaging was unavailable, the exact timing of tendon disruption and a definitive causal relationship between the trauma and tendon rupture cannot be established with certainty.

The physical examination was important in identifying pathology beyond the structural abnormality. Loss of the medial longitudinal arch, impaired tibialis posterior function, and inability to perform a single-leg heel rise indicated significant loss of dynamic medial support. Similar clinical presentations have been described in pediatric patients with chronic PTT deficiency, in whom tendon dysfunction may present with acquired flatfoot and impaired heel-rise function. In this patient, these findings provided clinical evidence of significant PTT dysfunction contributing to the progressive planovalgus deformity [11]. MRI subsequently clarified the additional tendon pathology. Nonvisualization of the PTT at the ankle level, together with tibialis posterior muscle atrophy, supported a chronic tendon lesion. MRI can provide additional information regarding soft-tissue structures in children with atypical or acquired flatfoot when the clinical findings cannot be fully explained by the initial structural assessment [12]. In the present case, the MRI findings, combined with the loss of tendon function and inability to perform a single-leg heel rise, strongly supported chronic complete PTT rupture. Intraoperative exploration further confirmed this diagnosis by demonstrating an empty PTT tunnel, a collapsed distal sheath, and absence of identifiable proximal or distal tendon remnants. The chronic nature of the lesion directly influenced surgical management. Because no suitable tendon stump could be identified, primary repair was not feasible. FHL tendon transfer was therefore selected to substitute for the absent PTT and restore dynamic medial support. FHL transfer has been successfully used for pediatric pes planus secondary to PTT injury and represents a suitable reconstructive option when chronic tendon deficiency prevents direct repair. Similar favorable outcomes have been reported in children and adolescents with acquired flatfoot and chronic PTT insufficiency treated with FHL transfer or tendon reconstruction [9,10,13].

Tendon reconstruction alone, however, would not necessarily correct the established multiplanar planovalgus deformity. The surgical procedure therefore addressed both the tendon deficiency and the associated structural malalignment. Triple percutaneous tendo-Achilles lengthening addressed the equinus component, while the 10-mm medial displacement calcaneal osteotomy corrected the hindfoot valgus. The 10-mm lateral column lengthening addressed the planovalgus component and associated forefoot malalignment. Lateral column lengthening is an established reconstructive technique for adolescent pes planovalgus and is used to improve overall foot alignment [14]. Thus, the procedure combined restoration of dynamic medial support with correction of the established bony deformity rather than relying on tendon reconstruction alone. At three years and nine months of follow-up reconstruction, the patient remained pain-free and had returned to near-normal walking and daily activity. Clinical assessment demonstrated satisfactory heel alignment and near-normal restoration of the medial longitudinal arch. Importantly, the patient regained the ability to perform a single-leg heel rise, which had been impossible before surgery. These findings indicate satisfactory restoration of dynamic medial support and maintenance of the correction over the available follow-up period.

Several limitations should be acknowledged. First, early post-traumatic imaging documenting an acute PTT rupture was unavailable. Although the patient had no documented pre-injury foot deformity, PTT dysfunction, or related complaints and subsequently developed progressive planovalgus with chronic PTT deficiency after the reported injury, the exact timing of tendon disruption and a definitive causal relationship between the trauma and tendon rupture cannot be established with certainty. Second, preoperative radiographs could not be retrieved from the hospital imaging system because of the prolonged interval since the initial treatment; therefore, standardized quantitative radiographic parameters, including calcaneal pitch and other angular measures of the hindfoot and medial column, could not be reliably compared between the preoperative and postoperative states. Third, validated patient-reported outcome measures, such as the Foot and Ankle Outcome Score, American Orthopaedic Foot & Ankle Society score, or a pediatric foot and ankle functional score, were not prospectively collected. Functional outcome was therefore assessed primarily through clinical examination, pain, activity level, return to daily, school, recreational, and sports activities, and restoration of single-leg heel-rise function. Finally, this is a single case, and the favorable long-term outcome should not be generalized to all pediatric patients with chronic PTT rupture or planovalgus deformity. Nevertheless, the case highlights the importance of correlating the clinical examination with appropriate imaging when evaluating progressive or acquired planovalgus following trauma, particularly when PTT dysfunction is present.

## Conclusions

PTT rupture is a rare but important cause of acquired planovalgus deformity in children and adolescents and should be considered when progressive unilateral flatfoot develops following trauma. Careful clinical examination, supported by MRI, can help identify chronic PTT insufficiency and guide appropriate surgical planning. When chronic tendon disruption and retraction make primary repair unfeasible, flexor hallucis longus tendon transfer may provide an effective alternative for restoring dynamic medial support. In patients with established multiplanar deformity, tendon reconstruction should be combined with appropriate correction of the associated bony malalignment to achieve a balanced and functional foot. In this case, the combined reconstructive procedure resulted in sustained pain relief, improved foot alignment, restoration of single-leg heel-rise ability, and return to daily activities over the long term. Although these favorable findings are encouraging, further reports are needed to clarify optimal management strategies and long-term outcomes for this rare condition in the pediatric population.
